# Metastatic Colorectal Cancer Beyond Third-Line Treatment: Systematic Review and Meta-Analysis of Later-Line Therapies

**DOI:** 10.3390/jcm15083128

**Published:** 2026-04-20

**Authors:** Jacopo Giuliani, Emilia Durante, Daniela Mangiola, Andrea Georgopulos, Beatrice Mantoan, Francesco Fiorica

**Affiliations:** 1 Department of Clinical Oncology, Section of Medical Oncology, AULSS 9 Scaligera, 37045 Legnago, Italy; 2 Department of Clinical Oncology, Section of Radiation Oncology and Nuclear Medicine, AULSS 9 Scaligera, 37045 Legnago, Italy; 3 Department of Diagnostic Imaging, AULSS 9 Scaligera, 37045 Legnago, Italy

**Keywords:** metastatic colorectal cancer, late-line therapy, trifluridine/tipiracil, regorafenib, fruquintinib, overall survival, systematic review

## Abstract

**Objectives**: To evaluate and indirectly compare overall survival (OS) and safety of regorafenib, fruquintinib, and trifluridine/tipiracil (TAS-102) monotherapy in refractory metastatic colorectal cancer (mCRC) beyond the third line. **Methods**: A systematic review and meta-analysis of phase II/III randomized controlled trials was conducted according to PRISMA guidelines. PubMed/MEDLINE, Embase, and Cochrane CENTRAL were searched from inception. Eligible studies included patients with mCRC previously treated with standard chemotherapy and biologic agents, receiving regorafenib, fruquintinib, or TAS-102 as monotherapy in the fourth line or later. OS data were reconstructed from published Kaplan–Meier curves. Pooled median and mean OS were estimated using a random-effects model, and heterogeneity was assessed using the I^2^ statistic. Safety outcomes were descriptively summarized. **Results**: Four RCTs were included. The pooled median OS was 7.83 months (95% CI: 6.98–8.80), and the pooled mean OS was 8.90 months (95% CI: 8.00–9.81), with no heterogeneity (I^2^ = 0%). Survival gains versus placebo ranged from 1.4 to 2.6 months. Survival curves largely overlapped, with differences below one month. Safety was consistent with known profiles. **Conclusions**: These agents provide comparable efficacy with modest survival benefit in late-line mCRC, highlighting the need for improved strategies and better treatment sequencing.

## 1. Introduction

Metastatic colorectal cancer (mCRC) remains a major cause of cancer-related mortality worldwide despite improvements in systemic therapies. In selected patients, tailored multidisciplinary approaches, including surgical resection of metastatic lesions, may offer long-term survival benefits, particularly in oligometastatic disease. Standard first- and second-line treatments typically consist of combination chemotherapy (fluoropyrimidine, oxaliplatin, irinotecan) with targeted agents such as anti-VEGF or anti-EGFR antibodies, achieving median overall survival (OS) approaching 30 months in selected populations. However, most patients eventually experience disease progression, and treatment options beyond the second line are limited and associated with modest benefit, underscoring an unmet clinical need in this refractory setting.

Two oral agents, trifluridine/tipiracil [1] (TAS102) and regorafenib [2], have long been established as standard salvage therapies for mCRC after progression on standard regimens, demonstrating statistically significant albeit incremental OS benefits compared with best supportive care (BSC) or placebo in pivotal trials. These agents are approved for use in patients previously treated with fluoropyrimidine-, oxaliplatin-, and irinotecan-based chemotherapy, as well as prior anti-VEGF therapy, and anti-EGFR therapy if RAS wild-type. Regorafenib, an oral multi-kinase inhibitor that targets angiogenic, stromal and oncogenic kinases including VEGFR, has been shown to increase OS in refractory mCRC and remains a benchmark in this setting.

More recently, combination strategies aiming to enhance efficacy over monotherapy have been investigated. The SUNLIGHT phase III trial [3] established that TAS102 in combination with bevacizumab (an anti-VEGF antibody) significantly improves survival outcomes versus (vs.) TAS102 alone in refractory mCRC, resulting in regulatory approvals in multiple jurisdictions for patients who have received at least two prior lines of therapy. In this study, the addition of bevacizumab increased median OS and progression-free survival (PFS) compared with TAS102 alone, supporting maintenance of anti-angiogenic blockade beyond progression on prior bevacizumab-containing regimens.

In parallel, regorafenib continues to be utilized as a salvage option, including after progression on TAS102 plus bevacizumab, with retrospective real-world analyses suggesting similar efficacy and safety outcomes in this sequence [4,5]. Additionally, fruquintinib, a highly selective oral inhibitor of VEGFR-1, -2 and -3, has emerged as an effective third-line therapy, demonstrating significant improvements in OS and PFS compared with placebo in the international phase III FRESCO-2 trial [6], with a median OS of 7.4 months in the fruquintinib group vs. 4.8 months in the placebo group (HR = 0.66, 95% CI 0.55–0·80, *p* < 0.0001). These results have informed regulatory approvals globally for previously treated mCRC, including in patients who have progressed following TAS102 and/or regorafenib.

Importantly, despite these advances, the absolute magnitude of survival benefit observed with currently available later-line therapies remains limited, typically below three months. This raises critical questions regarding their clinical relevance, cost-effectiveness, and overall value, particularly in heavily pretreated patients with limited life expectancy.

The optimal management of mCRC beyond the third line of therapy remains an area of substantial clinical uncertainty. While combination strategies such as TAS102 plus bevacizumab have reshaped the standard third-line approach, treatment decisions after failure of standard chemotherapy doublets and biologic agents and particularly after progression on a third-line regimen are not guided by direct comparative evidence. In routine practice, patients who have already received fluoropyrimidine, oxaliplatin and irinotecan-based chemotherapy, as well as anti-VEGF and, where appropriate, anti-EGFR therapy, may receive sequential oral agents including regorafenib, fruquintinib, or trifluridine/tipiracil (TAS-102) monotherapy, depending on prior sequencing strategies.

In clinical scenarios where TAS102 plus bevacizumab is administered in the third line, subsequent options are typically limited to regorafenib or fruquintinib. Conversely, in settings where regorafenib is chosen as third-line therapy, TAS-102 monotherapy may be reserved for later lines. Therefore, a clear understanding of the relative efficacy and safety of these agents beyond third-line therapy is critically needed to inform evidence-based sequencing decisions.

The primary aim of this study was to conduct a systematic review and quantitative meta-analysis of phase II/III randomized controlled trials (RCTs) with reconstructed survival data in order to evaluate OS among patients receiving regorafenib, fruquintinib, or TAS-102 monotherapy beyond the third line for refractory mCRC. Specifically, this analysis aimed to estimate pooled survival outcomes and to perform an indirect descriptive comparison of survival dynamics across approved late-line therapies in the absence of head-to-head trials.

By focusing exclusively on treatment beyond the third line and excluding combination strategies such as TAS-102 plus bevacizumab administered in third line, this meta-analysis aims to clarify the comparative benefit-risk profiles of currently available oral agents in the late-line setting. The results are expected to provide clinically meaningful evidence to guide sequencing strategies in heavily pretreated mCRC patients and to identify areas where prospective comparative trials are urgently needed.

## 2. Materials & Methods

### 2.1. Study Design

This study was designed as a systematic review and quantitative meta-analysis [7,8] conducted in accordance with the Preferred Reporting Items for Systematic Reviews and Meta-Analyses (PRISMA) guidelines [9,10]. In the Supplementary Material, you can find the PRISMA checklist [10].

In addition to OS, qualitative information on study characteristics, including inclusion and exclusion criteria, patient populations, and sample size assumptions, was collected to improve contextual interpretation of results.

Studies were included if they met the following criteria:

1. Population: adult patients (≥18 years) with histologically confirmed metastatic colorectal adenocarcinoma previously treated with at least three prior systemic lines, including fluoropyrimidine, oxaliplatin, irinotecan, and anti-VEGF therapy (and anti-EGFR therapy for RAS wild-type tumors when applicable).
2. Intervention: regorafenib, fruquintinib, or TAS-102 administered as monotherapy in the fourth line or later.
3. Comparator: placebo or best supportive care.
4. Outcomes: reporting OS with available Kaplan–Meier survival curves.

Retrospective studies, single-arm trials, non-randomized studies, third-line combination regimens, and non-English publications were excluded.

The primary and sole quantitative endpoint of this meta-analysis was OS, defined as the time from randomization to death from any cause.

Studies evaluating TAS-102 in combination with bevacizumab as third-line therapy were excluded, as the objective of the present analysis was to focus exclusively on treatments administered beyond the third line. Retrospective studies, single-arm trials without a comparator arm, case reports, and non-English publications were excluded.

Exclusion criteria were consolidated into a single section to avoid redundancy and improve clarity.

No time restrictions were applied in the literature search.

### 2.2. Search Strategy and Data Extraction

A comprehensive literature search was conducted in PubMed/MEDLINE, Embase, and the Cochrane Central Register of Controlled Trials (CENTRAL) from database inception to the most recent date prior to analysis. Major oncology conference proceedings were also screened. The search strategy combined terms related to metastatic colorectal cancer, refractory disease, late-line therapy, regorafenib, fruquintinib, TAS-102, randomized, and phase III trials. Reference lists of relevant articles were manually reviewed to identify additional eligible studies. The search strategy included combinations of the following keywords and Medical Subject Headings (MeSH) terms: “metastatic colorectal cancer,” “refractory colorectal cancer,” “fourth line,” “later line,” “regorafenib,” “fruquintinib,” “trifluridine,” “tipiracil,” “TAS-102,” “randomized,” and “phase III.”

The full search strategy (including complete search strings for each database) was defined to ensure reproducibility.

Two independent reviewers (JG and FF) screened titles and abstracts, followed by full-text evaluation according to predefined eligibility criteria. Discrepancies were resolved through discussion. From each eligible trial, the following study-level data were extracted: study name, publication year, geographic region, number of participants, treatment arms, and reported survival outcomes. Kaplan–Meier curves for OS were retrieved for reconstruction.

When individual patient data were not available, published Kaplan–Meier survival curves were digitized to reconstruct time-to-event data using established statistical methods. Extracted information was used exclusively for survival reconstruction and pooled survival estimation.

Information on planned sample size and statistical power calculations, when available, was also extracted.

Risk of bias was assessed independently by two reviewers using the Cochrane Risk of Bias tool, evaluating randomization, allocation concealment, blinding, completeness of outcome data, and selective reporting [11].

Publication bias was not formally assessed due to the limited number of included studies; this was clearly distinguished from study-level risk of bias assessment.

### 2.3. Statistical Analyses

Statistical analyses focused primarily OS.

Kaplan–Meier curves were digitized and individual patient data were reconstructed using the Guyot method. Numbers at risk were extracted when available, censoring was handled according to established methods, and reconstructed curves were visually validated against published data. Median OS and hazard ratios were considered primary reference endpoints, while mean OS was estimated to allow pooled survival curve generation and comparison of survival dynamics over time.

A random-effects model was used to account for potential between-study variability. Median OS values were summarized descriptively across studies and aggregated to provide an overall estimate.

Between-study heterogeneity was assessed using the I^2^ statistic and the χ^2^ test. An I^2^ value < 25% was considered low heterogeneity, 25–50% moderate, and >50% high heterogeneity.

Reconstructed survival curves were visually compared to evaluate differences in survival dynamics over time [12]. All statistical analyses were performed using R software R version 4.2.0 (R Foundation for Statistical Computing, Vienna, Austria), with survival and meta-analysis packages.

Given the limited number of included trials (n = 4), formal assessment of publication bias was not performed.

## 3. Results

### 3.1. Study Characteristics

A dedicated Study Characteristics section was introduced to improve clarity.

**Figure 1:**
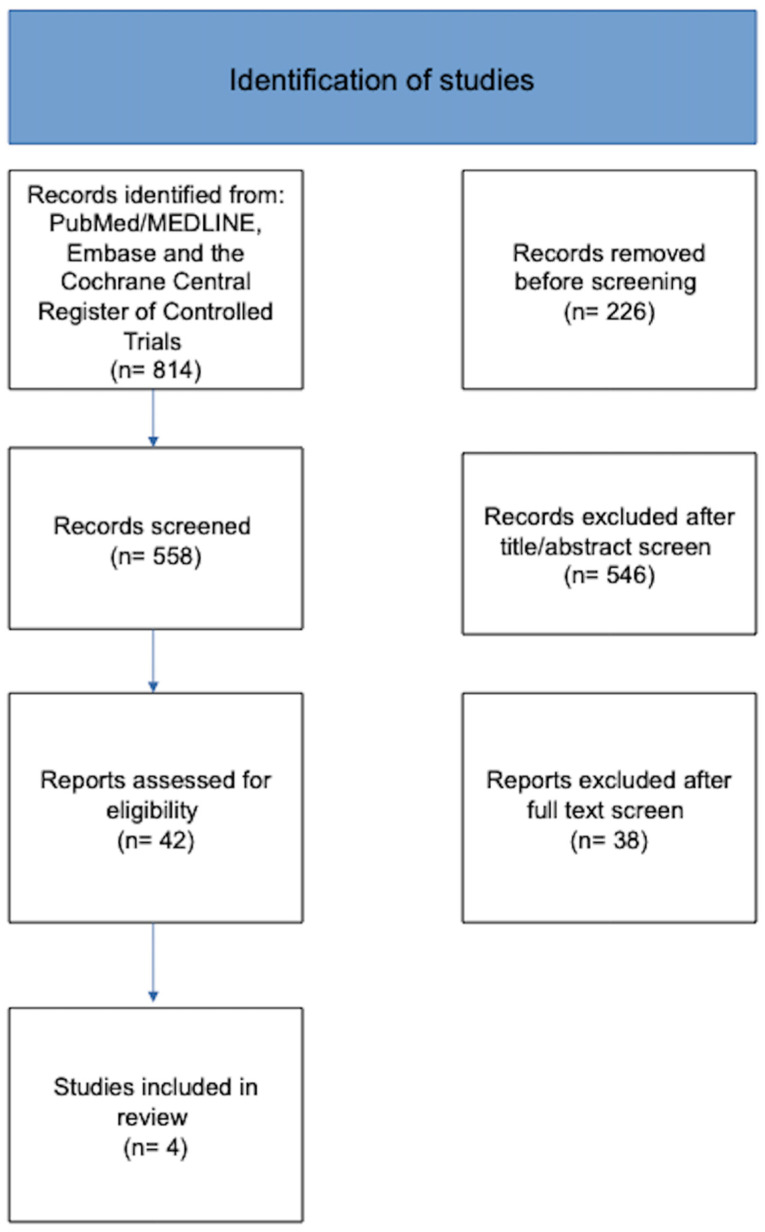
Study flow chart.

Four RCTs treatment were included in the meta-analysis (Figure 1). The investigated agents were TAS-102 alone (1 RCT), regorafenib (2 independent RCTs), and fruquintinib (1 RCT). All studies enrolled heavily pretreated patients with refractory disease and reported OS as the primary endpoint. The summary of the analyzed phase III RCTs is reported in Table 1.

Although inclusion criteria were broadly similar, differences were observed in geographic distribution and prior treatment exposure. Notably, the CONCUR trial [13] enrolled exclusively Asian patients and included a lower proportion of prior biologic therapy compared with other studies.

### 3.2. Overall Survival

#### Pooled Survival Estimates

The aggregated median OS across the included studies was 7.83 months (95% CI: 6.98–8.80 months). The pooled estimated mean OS was 8.90 months (95% CI: 8.00–9.81 months). No statistical heterogeneity was observed: I^2^ = 0%; this indicates a high degree of consistency in survival outcomes across the included RCTs.

The median OS gain versus placebo ranged from 1.4 to 2.6 months, with an approximate pooled improvement of 2.1 months.

No statistical heterogeneity was observed (I^2^ = 0%); however, this finding should be interpreted cautiously given the small number of included trials.

### 3.3. Comparative Analysis of Mean Overall Survival

Numerical differences in mean OS were observed among treatments:

- regorafenib (CONCUR) [13] showed the highest mean OS (9.34 months).
- TAS-102 (RECOURSE) [1] demonstrated a mean OS of approximately 9.07 months.
- regorafenib (CORRECT) [2] showed a mean OS of approximately 8.67 months.
- fruquintinib (FRESCO-2) [6] demonstrated a mean OS of approximately 8.50 months.

The maximum absolute difference between treatments remained below one month, indicating limited clinically meaningful differences across agents.

A summary of median OS, median PFS, and major grade ≥ 3 adverse events for each included trial is reported in Table 2, providing a direct comparison of efficacy and safety across approved late-line therapies.

### 3.4. Survival Curve Comparison

The reconstructed OS curves (Figure 1) demonstrated a broadly overlapping pattern across the four treatment strategies. At early time points (3–6 months), survival probabilities were comparable across agents, with slight numerical separation favoring regorafenib (CONCUR) [13]. Between 9 and 12 months, modest divergence was observed, with regorafenib (CONCUR) [13] maintaining the highest survival probability, followed by TAS-102 (RECOURSE) [1], while regorafenib (CORRECT) [2] and fruquintinib (FRESCO-2) [6] showed slightly lower but largely overlapping survival estimates. At later time points (15–18 months), survival probabilities converged, with all treatments demonstrating similar tail behavior and no evidence of substantial long-term divergence.

Overall, the survival curves indicate: parallel decline over time, absence of late separation and no visually appreciable heterogeneity in survival dynamics

**Figure 2:**
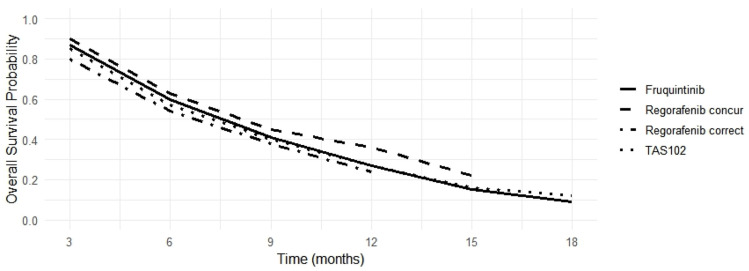
Overall survival probability over time according to treatment. Estimated overall survival probabilities at 3, 6, 9, 12, 15, and 18 months for patients treated with fruquintinib, regorafenib (CONCUR trial [13] and CORRECT trial [2]) and TAS-102.

These findings are consistent with the quantitative heterogeneity analysis (I^2^ = 0%), supporting comparable survival benefit across approved fourth-line and later therapies in refractory mCRC (Figure 2).

## 4. Discussion

This meta-analysis of pivotal randomized phase III trials demonstrates that currently approved later-line therapies for mCRC provide consistent survival outcomes across studies, with a pooled median overall survival of 7.83 months and a mean overall survival of 8.90 months. The absence of statistically detectable heterogeneity (I^2^ = 0%) suggests a high degree of consistency across trials; however, this finding should be interpreted cautiously given the limited number of included studies and the known low statistical power of heterogeneity metrics in small meta-analyses.

Despite this apparent consistency, the most clinically relevant finding of the present analysis is the limited magnitude of benefit observed across all included treatments. The median overall survival gain versus placebo ranged from 1.4 to 2.6 months, with an approximate pooled improvement of 2.1 months. While these differences reached statistical significance in all trials, their clinical meaningfulness remains debatable, particularly in a population characterized by advanced disease, cumulative treatment toxicity, and limited life expectancy.

This discrepancy between statistical significance and clinical relevance is a well-recognized challenge in oncology drug development, especially in later-line settings. In heavily pretreated patients, even small survival gains may be considered valuable; however, such benefits must be carefully contextualized within a broader framework that includes quality of life, symptom control, and treatment burden.

From a mechanistic perspective, the agents included in this analysis share overlapping anti-angiogenic or cytotoxic properties, which may partially explain the similarity in efficacy outcomes. Regorafenib and fruquintinib both target angiogenic pathways through VEGFR inhibition, while TAS-102 exerts cytotoxic effects via nucleoside analog incorporation. The convergence of survival curves observed in this analysis suggests that these different mechanisms ultimately translate into comparable clinical outcomes in the refractory setting, where tumor biology may be dominated by resistance mechanisms and clonal heterogeneity.

An important aspect that warrants careful consideration is the toxicity profile of these agents. Although not quantitatively analyzed in this meta-analysis, previous studies have consistently reported clinically relevant adverse events associated with later-line therapies. Regorafenib and fruquintinib are commonly associated with hypertension, hand-foot skin reaction, fatigue, and hepatotoxicity, while TAS-102 is associated with hematologic toxicities such as neutropenia and anemia. These adverse events may significantly impact treatment adherence, dose intensity, and patient quality of life, and should therefore be integrated into treatment decision-making.

In addition to toxicity, the economic burden of later-line therapies represents a critical and often under-discussed issue. The relatively high cost of these oral agents, combined with modest survival benefits, raises concerns regarding cost-effectiveness and sustainability, particularly in healthcare systems with limited resources. In this context, the value of treatment should not be assessed solely on statistical endpoints, but rather on a comprehensive evaluation of clinical benefit, toxicity, patient-reported outcomes, and economic impact.

Another key consideration relates to the design of the included trials, all of which used placebo as the comparator. While this approach allows for clear demonstration of drug activity, it may overestimate the perceived benefit in the absence of active comparators. In real-world clinical practice, patients often receive sequential therapies, and the relative positioning of these agents remains uncertain. Therefore, cross-trial comparisons should be interpreted with caution, and the lack of head-to-head studies represents a significant gap in the current evidence base.

The interpretation of numerical differences between trials is further complicated by differences in study populations. In particular, the CONCUR trial enrolled exclusively Asian patients and included a lower proportion of patients previously treated with biologic agents compared with the CORRECT trial. These differences in baseline characteristics, prior treatment exposure, and potential pharmacogenomic factors may partially explain the numerically higher survival observed in CONCUR. This highlights the importance of considering population heterogeneity when interpreting indirect comparisons.

From a methodological standpoint, this study employed reconstruction of individual patient data from published Kaplan–Meier curves, which represents a well-established approach in the absence of original datasets. To enhance the robustness of the analysis, numbers at risk were incorporated when available, censoring was handled according to standard methods, and reconstructed curves were visually validated against published data. Nevertheless, this approach remains inherently limited and cannot fully substitute for analyses based on original individual patient data.

The use of mean overall survival derived from reconstructed data represents an additional methodological consideration. While median OS remains the standard endpoint in oncology trials, mean OS provides complementary information by capturing the entire survival distribution and enabling the generation of pooled survival curves. This approach allows for a more comprehensive comparison of survival dynamics over time, although it should be interpreted alongside conventional endpoints.

The absence of statistically significant heterogeneity should not be interpreted as definitive evidence of equivalence between treatments. Given the small number of included studies, both I^2^ and χ^2^ tests have limited sensitivity to detect true heterogeneity. Therefore, the observed consistency in outcomes should be viewed as suggestive rather than conclusive.

Importantly, the findings of this analysis reinforce the concept that treatment selection in the later-line setting should be individualized. Given the minimal differences in efficacy, factors such as toxicity profile, patient comorbidities, performance status, prior treatment exposure, route of administration, and patient preferences should play a central role in decision-making.

The modest survival gains observed also raise the question of potential clinical futility in certain subgroups of patients, particularly those with poor performance status or rapidly progressive disease. In such cases, best supportive care or palliative approaches may represent more appropriate options, emphasizing the need for careful patient selection and shared decision-making.

This analysis has several limitations. First, it is based on reconstructed rather than original individual patient data, limiting the ability to perform adjusted analyses. Second, cross-trial comparisons are inherently subject to bias due to differences in study design and patient populations. Third, the small number of included studies limits the robustness of statistical inferences, particularly with respect to heterogeneity and publication bias. Fourth, toxicity and quality of life outcomes were not quantitatively assessed, representing an important area for future research.

Finally, despite the availability of multiple later-line therapies, the overall prognosis of patients with refractory mCRC remains poor, underscoring a substantial unmet clinical need [14]. Future research should focus on biomarker-driven approaches, identification of predictive factors of response, rational combination strategies, and the development of more effective treatments. Prospective head-to-head trials and real-world studies will be essential to better define optimal sequencing strategies and improve patient outcomes.

## 5. Conclusions

In this meta-analysis of pivotal phase III RCTs evaluating TAS-102, regorafenib, and fruquintinib in refractory mCRC, survival outcomes were highly consistent, with no statistical heterogeneity and only modest numerical differences between agents. Although statistically significant, the survival benefits observed with currently available later-line therapies remain modest and of uncertain clinical relevance, with median improvements consistently below three months. These findings highlight the importance of integrating toxicity, quality of life, cost-effectiveness, and patient preferences into treatment decisions, as well as the urgent need for more effective therapeutic strategies.

## Figures and Tables

**Table 1:** Summary of phase III RCTs evaluating fruquintinib, regorafenib, and TAS-102 in patients with metastatic colorectal cancer. For each study (FRESCO-2, CORRECT, CONCUR, and RECOURSE), the table reports the treatment regimen, total number of enrolled patients (N), primary endpoint, median overall survival (OS), absolute OS gain compared with placebo, and corresponding *p*-value.

Trial	Comparative Regimens	Total N Patients	Primary Endpoint	Median OS (Months)	OS Gain (Months)	*p*-Value	Sample Size/Power	Key Inclusion Criteria
FRESCO-2 [6]	fruquintinib 5 mg/day (D1–D21, q28d)	461	OS	7.4	2.6	**<0.0001**	**Sample size powered for OS with HR 0.66**	Adults ≥ 18 y, mCRC previously treated with ≥3 lines including fluoropyrimidine, oxaliplatin, irinotecan, and anti-VEGF
	placebo	230		4.8				
CORRECT [2]	Regorafenib 160 mg/day (D1–D21, q28d)	505	OS	6.4	1.4	**0.0050**	Powered to detect HR 0.70 with 80% power	Adults ≥ 18 y, mCRC refractory to standard therapies
	placebo	255		5.0				
CONCUR [13]	Regorafenib 160 mg/day (D1–D21, q28d)	136	OS	8.8	2.5	**<0.001**	Planned sample size based on OS HR 0.55	Asian adults ≥ 18 y, mCRC previously treated with ≥2 lines chemotherapy; ECOG PS 0–1
	placebo	68		6.3				
RECOURSE [1]	TAS-102 35 mg/m^2^ BID (D1–D5 and D8–D12, q28d)	534	OS	7.1	1.8	**<0.001**	Powered for OS with 80% power	Adults ≥ 18 y, mCRC refractory to ≥2 prior regimens including fluoropyrimidine, oxaliplatin, irinotecan, anti-VEGF; RAS wild-type for anti-EGFR
	placebo	266		5.3				

Legend: N = number; OS = overall survival, TAS-102 = trifluridine/tipiracil; ECOG PS = Eastern Cooperative Oncology Group Performance Status. OS Gain = absolute median OS improvement vs. placebo. Key inclusion/exclusion criteria are summarized for clarity. Sample size/power details reported when available.

**Table 2:** Comparative Efficacy and Safety of Late-Line Therapies in Metastatic Colorectal Cancer.

Trial	Median OS (Months)	Median PFS (Months)	Major Grade ≥ 3 Adverse Events	Clinical Relevance/Comments
FRESCO-2 [6]/fruquintini	7.4	3.7	Hypertension 10%, Hand-foot syndrome 6%, Fatigue 5%	Modest OS benefit (2.6 months vs. placebo), manageable toxicity; clinically relevant for patients refractory to prior TAS-102/regorafenib.
CORRECT [2]/regorafenib	6.4	1.9	Fatigue 9%, Hand-foot syndrome 17%, Hypertension 7%	Incremental OS improvement (1.4 months vs. placebo) with notable toxicities; requires careful patient selection.
CONCUR [13]/regorafenib	8.8	3.2	Fatigue 13%, Hand-foot syndrome 21%, Hypertension 9%	Numerically higher OS (2.5 months vs. placebo) in Asian population; toxicity profile similar to CORRECT; clinical benefit modest but consistent.
RECOURSE [1]/TAS-102	7.1	2.0	Neutropenia 38%, Anemia 10%, Fatigue 5%	OS gain of 1.8 months vs. placebo; hematologic toxicity is most relevant; clinical benefit limited but reproducible.

Legend: OS = Overall Survival; PFS = Progression-Free Survival; Grade ≥ 3 AEs = Common high-grade toxicities; Clinical relevance includes magnitude of OS benefit and safety considerations.

## Data Availability

The original contributions presented in this study are included in the article/Supplementary Material. Further inquiries can be directed to the corresponding author.

## Supplementary Materials

The following supporting information can be downloaded at: https://www.mdpi.com/article/10.3390/jcm15083128/s1, PRISMA 2020 checklist.
